# Supplementary material for: Regulation of Decay Accelerating Factor Primes Human Germinal Center B Cells for Phagocytosis
Source: Front Immunol. 2021 Jan 5;11:599647. doi: 10.3389/fimmu.2020.599647 (PMC7813799; doi:10.3389/fimmu.2020.599647)
Supplement: Supplementary file 1 [file DataSheet_2.pdf]

Supplemental figure S1

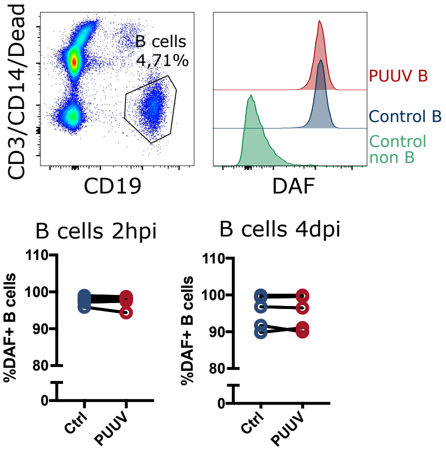

Supplemental figure S1.

In vitro incubation of PBMCs with PUUV Hantavirus and expression of DAF post-incubation

Supplemental figure S2

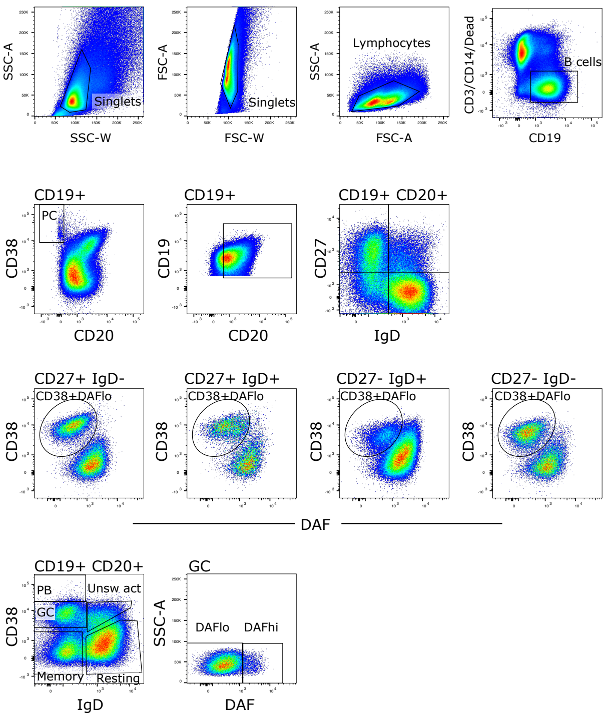

Supplemental figure S2. Gating strategies for analysis of human tonsillar B cells

Supplemental figure S3

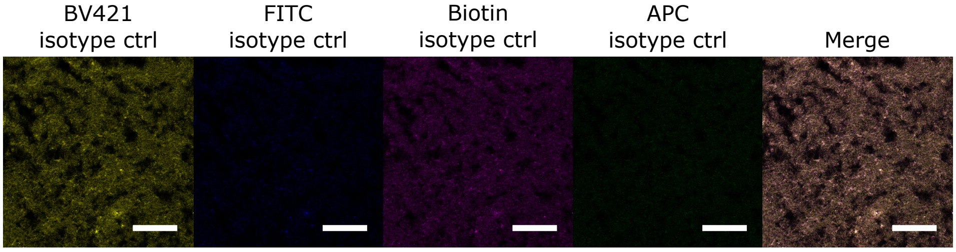

Figure S3. Fluorescent immuno stainings of human tonsil with conjugated non-specific antibodies. 20X magnification.

Supplemental Figure S4

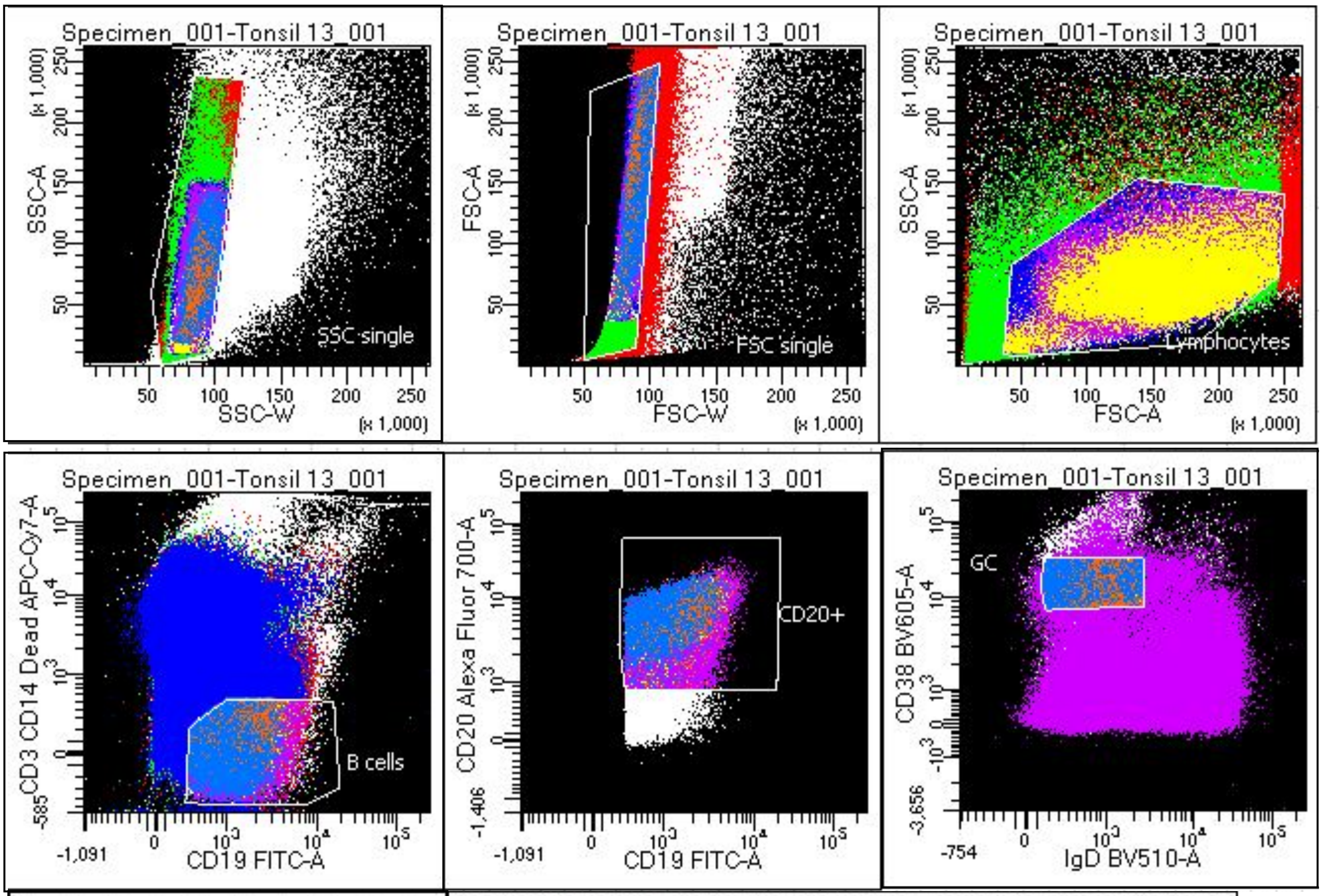

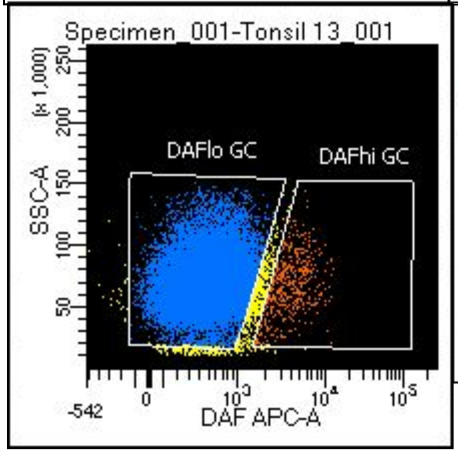

Tube: Tonsil 13_001

Population	#Events	%Parent	%Total
All Events	1,130,350	####	100.0
SSC single	1,055,939	93.4	93.4
FSC single	1,016,634	96.3	89.9
Lymphocytes	987,805	97.2	87.4
B cells	324,342	32.8	28.7
CD20+	313,392	96.6	27.7
GC	24,292	7.8	2.1
DAFlo GC	23,039	94.8	2.0
DAFhi GC	477	2.0	0.0

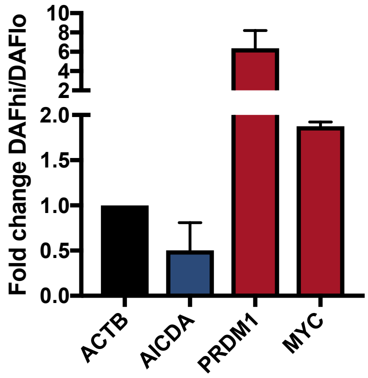

Supplemental Figure S4. Gating strategy for sorted DAFhi and DAFlo GC B cells. Validation of selected differentially expressed genes by qPCR.
