# Supplementary material for: Regulation of Decay Accelerating Factor Primes Human Germinal Center B Cells for Phagocytosis
Source: Front Immunol. 2021 Jan 5;11:599647. doi: 10.3389/fimmu.2020.599647 (PMC7813799; doi:10.3389/fimmu.2020.599647)
Supplement: Supplementary file 2 [file Table_1.pdf]

Table S1. List of anti-human antibodies used in flow cytometry.

Surface molecule	Fluorochrome	Clone	Manufacturer
Blimp1	PE	6D3	BD Biosciences
CD3	APC-Cy7	SK7 (Leu4)	BD Biosciences
CD10	APC	HI10a	BD Biosciences
CD10	PE	HI10a	Biolegend
CD14	APC-H7	HCD14	Biolegend
CD19	FITC	HIB19	BD Biosciences
CD19	APC-R700	HIB19	BD Biosciences
CD19	PE-Cy7	J3-119	Beckman Coulter
CD20	Alexa Fluor 700	2H7	Biolegend
CD20	BB700	2H7	BD Biosciences
CD20	Pacific Blue	2H7	Biolegend
CD21	PE-Cy7	B-ly4	BD Biosciences
CD27	BV421	M-T271	BD Biosciences
CD27	PE-Cy7	M-T271	BD Biosciences
CD34	PerCP-Cy5.5	8G12	BD Biosciences
CD35	BB515	E11	BD Biosciences
CD38	BV605	HIT2	Biolegend
CD38	APC-Alexa750	LS198-4-3	Beckman Coulter
CD45	Pacific Orange	HI30	Invitrogen
CD46	PE-Dazzle594	TRA-2-10	Biolegend
CD55	APC	JS-11	Biolegend
CD55	PE	JS-11	Biolegend
CD59	BV786	P282	BD Biosciences
CD71	PE-Cy5	M-A712	BD Biosciences
CD71	BV421	M-A712	BD Biosciences
CD83	BV421	HB15e	BD Biosciences
CD95	PE-CF594	DX2	BD Biosciences
CXCR4	Biotin	12G5	Biolegend
Fcrl5	PE	509f6	Biolegend
IgD	BV510	IA6-2	BD Biosciences
Ki67	PE-Dazzle594	Ki67	Biolegend
**Secondary reagents**			
Streptavidin	FITC	---	BD Biosciences
Streptavidin	PE-Cy5	---	BD Biosciences
