# Supplementary material for: Regulation of Decay Accelerating Factor Primes Human Germinal Center B Cells for Phagocytosis
Source: Front Immunol. 2021 Jan 5;11:599647. doi: 10.3389/fimmu.2020.599647 (PMC7813799; doi:10.3389/fimmu.2020.599647)
Supplement: Supplementary file 3 [file Table_2.pdf]

Table S2. List of anti-human antibodies used in fluorescent immunohistochemistry.

Surface molecule	Fluorochrome	Clone	Manufacturer
CD19	FITC	HIB19	BD Biosciences
CD55	APC	JS11	Biolegend
CXCR4	Biotin	12G5	Biolegend
IgD	BV421	IA6-2	BD Biosciences
**Secondary reagents**			
Streptavidin	PE-CF594	---	BD Biosciences

Isotype control	Fluorochrome	Clone	Manufacturer
Mouse IgG1 k	APC	---	Biolegend
Mouse IgG2A k	BV421	---	BD Biosciences
Mouse IgG2A k	Biotin	---	BD Biosciences
Mouse IgG2A k	FITC	---	BD Biosciences
**Secondary reagents**			
Streptavidin	PE-CF594	---	BD Biosciences
